# Biochemical and Physiological Plant Processes Affected by Seed Treatment with Non-Thermal Plasma

**DOI:** 10.3390/plants11070856

**Published:** 2022-03-23

**Authors:** Vida Mildaziene, Anatolii Ivankov, Bozena Sera, Danas Baniulis

**Affiliations:** 1 Faculty of Natural Sciences, Vytautas Magnus University, LT-44404 Kaunas, Lithuania; anatolii.ivankov@vdu.lt; 2 Department of Environmental Ecology and Landscape Management, Faculty of Natural Sciences, Comenius University in Bratislava, 84215 Bratislava, Slovakia; bozena.sera@uniba.sk; 3 Institute of Horticulture, Lithuanian Research Centre for Agriculture and Forestry, LT-54333 Babtai, Lithuania; danas.baniulis@lammc.lt

**Keywords:** non-thermal plasma, gene expression, germination, photosynthesis, phytohormones, plant yield, secondary metabolism, seeds, stress signal

## Abstract

Among the innovative technologies being elaborated for sustainable agriculture, one of the most rapidly developing fields relies on the positive effects of non-thermal plasma (NTP) treatment on the agronomic performance of plants. A large number of recent publications have indicated that NTP effects are far more persistent and complex than it was supposed before. Knowledge of the molecular basis and the resulting outcomes of seed treatment with NTP is rapidly accumulating and requires to be analyzed and presented in a systematic way. This review focuses on the biochemical and physiological processes in seeds and plants affected by seed treatment with NTP and the resulting impact on plant metabolism, growth, adaptability and productivity. Wide-scale changes evolving at the epigenomic, transcriptomic, proteomic and metabolic levels are triggered by seed irradiation with NTP and contribute to changes in germination, early seedling growth, phytohormone amounts, metabolic and defense enzyme activity, secondary metabolism, photosynthesis, adaptability to biotic and abiotic stress, microbiome composition, and increased plant fitness, productivity and growth on a longer time scale. This review highlights the importance of these novel findings, as well as unresolved issues that remain to be investigated.

## 1. Introduction

Research on plasma interaction with seeds is driven by the ever-increasing demand for food and other agricultural products in the context of scarce resources [1]. The development of new environmentally benign technologies for enhancing agricultural production is based on exploiting the natural adaptability of plants, and is essential for reducing unsustainable use of water, nutrients and agricultural chemicals [2]. The pre-sowing seed processing using different methods has been used to improve germination and seedling growth (reviewed in [3,4]). Recently, the use of plasma technologies for seed treatment has attracted increasing interest due to numerous positive effects reported on plant agricultural performance.

The first non-thermal atmospheric pressure plasma (NTP) sources were developed more than three decades ago [5]. Since then, the NTP technology has been used in various industries to modify the physico-chemical properties of treated substances in solid or liquid materials, as well as in the form of microparticles [6]. NTP offers a broad range of industrially interesting applications. The main advantage of plasma compared to other media is its ability to produce active energy-containing species that initiate physical changes and chemical reactions, which otherwise would not occur or proceed with difficulties [7]. Due to its technical and economic advantages, NTP treatment has been increasingly exploited for many practical purposes, such as sterilization, water purification, microfabrication, medicine, and agriculture.

NTP does not cause thermal damage to heat-sensitive biological systems such as living cells and tissues. Therefore, this technology has many applications in biomedical technologies [8,9,10,11,12], for o has shown potential to improve agronomic seed quality by surface decontamination, germination enhancement, and promotion of plant growth, as discussed in numerous reviews published recently [3,13,14,15,16,17,18,19,20]. Intensive research is currently being conducted on the application of NTP in agriculture, forestry, and food industries in many parts of the world. Despite numerous attempts, the molecular mechanisms underlying the effects of seed exposure to NTP remain elusive, and the published reviews do not cover the most recent experimental findings in sufficient detail.

Therefore, we aimed to provide an overview of the existing knowledge on changes in both biochemical and physiological processes, induced by seed treatment with NTP, expecting to aid in the understanding of this issue and to outline the possible development directions. The review consists of an introductory description of plasma as a complex agent that initiates multiple processes by interaction with seeds, followed by sections organized to distinguish the consequent stages in the complex response of plants to stress induced by NTP treatment, starting with the early changes in dry seeds, changes in germinating seeds, and changes observed in growing seedlings and plants. The effects of seed treatment with NTP on DNA methylation, wide-scale changes in gene and protein expression, enzyme activities in the affected seeds and growing plants are considered in the context of the effects observed in plant growth and yield. Particular attention is paid to the importance of seed dormancy, the role of reactive oxygen and nitrogen species and phytohormones, the mobilization of secondary metabolism, increased adaptability to stress, and the effects on the plant-associated microbiome.

## 2. Definitions of Plasma, Low-Temperature Plasma and Description of Different Types of Devices Used for Seed, Plant or Water Treatment

When sufficient heat is applied, solid material transforms into a liquid first and then, at a higher temperature, into a gas. As the energy supply increases, electrons receive sufficient energy to separate from atoms or molecules of gas and become electrically conductive. In this way, gas undergoes a transition to a partially or completely ionized gas, called the plasma state (physical plasma) [21]. Depending on the type of energy supplied and the amount of energy transferred to the plasma, plasma properties change in terms of electron density or temperature [22].

Physical plasma is the fourth state of matter, in which matter displays a behavior different from that observed in the other three states (solid, liquid, and gas). It is an electrically quasi-neutral gas with chemically reactive species such as electrons, ions, and neutrals [23]. Physical plasma is distinguished into high-temperature plasma (5 × 10^4^–10^6^ K) and low-temperature plasma (≤5 × 10^4^ K), denoted as LTP. LTP is subdivided into thermal plasma and NTP. Thermal plasmas are characterized by a thermodynamic equilibrium among free electrons, ions and neutrals [24].

On the other hand, the energy in NTP is supplied to free electrons only while the overall temperature of ions and neutrals remains significantly lower [5]. NTP contains charged particles (free electrons, ions) and neutral activated species, including gas molecules, free radicals, metastable particles and generated photons (including UV) [24]. The particles are not in a thermodynamic equilibrium; both ions and neutrals are near room temperature, whereas electrons are much hotter. This type of plasma is characterized by a strong thermodynamic non-equilibrium state, high selectivity, low gas temperature, and the presence of reactive chemical species. The temperature of NTP heavy particles (ions, molecules) is low, so they do not damage thermally sensitive materials, but their electrons reach sufficient energy to participate in plasma-chemical reactions. Therefore, NTP does not cause thermal damage to materials, but it is rich in various chemically reactive particles. NTP is used to treat heat-sensitive materials, such as human, animal, and plant tissue, hair, leather, wood, blood, various polymers and proteins [6,25,26].

In the laboratory, NTPs are often generated by electrical discharges in various gases, typically air, oxygen, helium, argon, nitrogen, or their mixtures. Typical discharges differ in electrode arrangement, power sources, gas pressure, and include glow discharge, plasma jets, low-pressure capacitively coupled plasma (CP) discharge, corona discharge, dielectric barrier discharge (DBD), diffuse coplanar surface barrier discharge (DCSBD), etc. (for details, see reviews [19,22,27,28,29,30]). In this review, the common abbreviation NTP is used for plasma generated by all types of devices.

Relatively low-level energy (2–5 eV) imparted to electrons initiates dissociation, excitation, and ionization reactions upon collision with gas atoms and molecules at a temperature close to ambient [31]. In the air atmosphere, it leads to the excitation of nitrogen (N_2_) molecules, dissociation of molecular oxygen (O_2_), and accumulation of ozone (O_3_) [32]. An increase in electron energies results in the dissociation of N_2_ and the production of nitrogen oxides (NO_x_) that inhibit ozone production and subsequently recombine to form several other reactive nitrogen species, including the oxidant and nitrating agent peroxynitrite anion (ONOO^−^). In the presence of water vapor, a hydroxyl radical (^•^OH) is produced by dissociation of water and by secondary processes involving neutralization of ions or by reactions of excited states of O_2_ and N_2_ [32].

The main biologically active component of NTP is a complex mixture of reactive oxygen and nitrogen species (ROS and RNS, combined abbreviation—RONS), such as superoxide anion (O_2_^•−^), NO, hydrogen peroxide (H_2_O_2_), ^•^OH, or ONOO^−^ that play important roles as signaling messengers in eukaryotic cells [33] and are involved in the regulation of seed dormancy, germination [33,34], and plant physiology [35].

The unique transfer of chemical reactivity and energy from gaseous plasmas to water occurs in the absence of any other chemicals, but results in a product with a notable transient broad-spectrum biological activity, referred to as plasma-activated water (PAW). These characteristics make PAW a friendly treatment for a wide range of biotechnology applications, including the agriculture and food industry [36]. Although PAW belongs to a broader topic of physical plasma (effects on plant biochemical and physiological processes reviewed recently in [16]), we will not address the issue of PAW in this text.

Thus, NTP is a complex physical stressor that can be applied for seed processing. The variation in plasma sources, specific plasma parameters, and protocols used for seed treatment represents one aspect (physical) of the difficulties in comparing the results obtained by various research groups. In turn, plant seeds are tiny reproductive plant structures that have all the features of complexity typical of biological systems. Therefore, research of interactions between such two systems is an extremely challenging task that requires systematic methods and novel fundamental findings to uncover the most important determinants of both the physical and biological aspects.

## 3. NTP Effects on Seed Germination and Early Seedling Growth

The first reports on the application of NTP in plant biology were published in 2000 and described the effects on seed germination induced by NTP treatment [37,38]. Dubinov et al. [37] treated oat (*Avena sativa*) and barley (*Hordeum vulgare*) seeds with air glow discharge for several minutes in both continuous and pulsed mode. The continuous mode stimulated seed germination more effectively than the pulsed mode but no changes in early growth of the seedlings were observed. Volin et al. [38] applied longer treatments (2–20 min) of low-pressure radio frequency (RF) rotating plasma in fluorocarbon or nitrogen or carbon-containing compound atmosphere for the treatment of barley (*Hordeum vulgare*), radish (*Raphanus sativus*), pea (*Pisum sativum*), soybean (*Glycine max*), corn (*Zea mays*), and bean (*Phaseolus vulgaris*) seeds, and observed strong negative effects on germination in the majority of cases. A relatively complex work focused on the physiological characteristics of light-induced seed germination affected by NTP was published in 2004 by Živkovič et al. [39]. The authors considered that the stimulation of seed germination of the empress tree (*Paulownia tomentosa*) with NTP could have been explained by three different physical mechanisms: etching, surface functionalization, and deposition of small bioactive molecules. Meiqiang et al. [40] treated tomato seeds with magnetized plasma (arc discharge combined with magnetic field) and observed no effects on germination in vitro. However, seedling emergence in pots was enhanced, and some of the treatment protocols resulted in increased activity of enzymes in seedling tissues (peroxidase in hypocotyls and dehydrogenase in roots), as well as an increase in the number of fruits and fruit biomass per plant. Strong stimulation of Lamb’s Quarters (*Chenopodium album* agg.) seed germination after using low-pressure microwave plasma treatment was explained by cracks found on the seed surface (electron microscope scanning), where water could better penetrate seeds [41]. Moreover, the experiment was carried out with dormant Lamb’s Quarters seeds, which germinate gradually under natural conditions for many decades, and a threefold increase in seed germination was obtained compared to the control under laboratory conditions, and seedling size was also significantly larger [42].

Since then, numerous studies have been published. The results of a large part of these studies are summarized in Table 1. Considering that germination test results may depend on the used method, only the reports representing in vitro germination tests (tests for *sensu stricto* [43] germination) are presented in Table 1. For brevity, we do not give Latin names of plant species in the following text. The in vitro germination test is typically performed in a Petri dish after seed imbibition on wet filter paper under controllable laboratory conditions (temperature, light, humidity). The number of germinated seeds is periodically counted by the appearance of visible radicles that protrude through the seed coat.

As shown in Table 1, the effects of seed treatment using different NTP devices and protocols have been extensively studied in a wide variety of plants including the main strategic crops (such as wheat, corn, legumes, and oilseeds). Stimulating effects of seed treatment on seed germination in vitro have been demonstrated in most of the publications [37,41,42,44,45,46,47,48,49,50,51,52,53,54,55,56,57,58,59,60,61,62,63,64,65,66,67,68,69,70,71,72,73,74,75,76,77,78,79,80,81,82,83,84,85,86,87,88,89,90,91,92,93,94,95,96,97,98,99,100,101,102,103,104,105,106,107,108]. Most of these reports represent studies limited to the effects on germination and early seedling growth (from 4 days to several weeks), indicating that these two aspects are commonly recognized as the main criteria for estimating plant response to NTP treatment. Neutral [49,64,70,76,92,105,107,109,110,111,112] or negative [35,47,72,81,105,110,112,113,114] effects were reported in a smaller number of studies. This might be explained by the limited chances of publishing negative or neutral results, although such data may also be relevant. Unfortunately, it is difficult to develop an overarching comparative study due to the different methods used to generate plasma and the treatment protocols, i.e., non-standardized treatments.

However, the effects of different equipment on the same seed lots have been compared in a few studies. The germination and early growth of buckwheat were estimated after treatment with low-temperature plasma discharge in air gas generated in 4 types of devices [105]. A positive effect on germination and early growth was observed after applying the gliding arc device, while a strong negative effect was induced by DBD plasma; seed treatment using apparatus with a planar rotating electrode or downstream microwave plasma caused slight inhibition of germination and growth. Such results indicate that treatment protocols must be carefully optimized for each piece of plasma equipment. A recent study compared the effects of three different plasma devices (RF plasma in vacuum, microwave-driven atmospheric-pressure plasma, DBD atmospheric pressure plasma) on corn yield in the field [115]. However, all devices were equally ineffective in their experiment. Air DBD discharge was more effective than the helium plasma jet used to stimulate *Arabidopsis thaliana* seed germination [86].

Different gases can be used for plasma generation (Table 1). Ambient air is used most often, but argon, oxygen, nitrogen, helium, or mixtures of several gases can also be applied. The chemical composition of the generated reactive plasma particles depends on the gaseous phase; therefore, studies comparing the effects of different feeding gases on seed germination are expected to reveal the reactive species responsible for the observed NTP effects. On the flip side, plasma density and UV radiation parameters, as well as the dose of energy transferred to the sample, also depend on the feeding gas [92,100,116].

**Table 1:** Effects of different plasma treatment equipment on plant germination in vitro.

Effect	NTP Device	Plant Species (NTP Feeding Gas if Not Air) [References]
Positive	Low-pressure CP	Ajwain [44], bean [45,46], black mulberry [47]; industrial hemp [48], lamb’s quarters [41,42], lentil [45], maize [46], mung bean [50], wheat [37,45,49], oilseed rape [52], quinoa [53], red clover [54,55], rice [56], soybean [57], sunflower [58], tomato [40,59]; artichoke (nitrogen) [60], common bean (oxygen) [61], safflower (argon) [62], wheat (helium) [63], wheat (argon) [64].
	DBD plasma	Barley [65], black pine [66], cotton [67], cucumber, [68], green chiretta [69], Norway spruce [70], quinoa [53]; pea [71,72], pepper [68], radish [73,74,75,76,77,78]; rice [79,80], sunflower [81,82], sweet basil [83], thale cress [84,85,86,87], wheat [51,88,89], zinnia [84]; barley (nitrogen + 0.65% air) [90], carrot (argon) [91], coriander (nitrogen) [92], cotton (argon) [67], rice (argon + air) [93], soybean (oxygen, nitrogen) [94,95], soybean (argon) [96], sweet basil (argon + oxygen) [97], wheat (argon/air, argon/oxygen) [98,99].
	Plasma jet	Common bean (helium) [100], *Erythrina velutina* (helium) [101], fenugreek (argon) [102] mung bean (air, oxygen) [103], wheat (nitrogen) [104].
	Gliding arc	Buckwheat (air) [105], garden tree-mallow (nitrogen) [106], industrial hemp (air) [107], maize (air) [108].
Neutral	Low-pressure CP	Blue lupin [49], buckwheat [109], wheat (oxygen) [110].
	Gliding arc	Buckwheat [105], industrial hemp [107].
	DBD plasma	Coriander [92], maize [111], Norway spruce [70], radish [76], Scotish pine [112].
	Plasma jet	Mung bean (helium, nitrogen) [64]
Negative	Low-pressure CP	Barley, radish, pea, soybean, corn, bean (fluorocarbon, nitrogen, carbon-containing compounds) [35], Norway spruce [113], rhododendron [47], buckwheat (oxygen) [114], wheat (oxygen) [110].
	DBD plasma	Buckwheat [105]; Scots pine [112], pea (nitrogen, oxygen) [72], sunflower [81].

DBD plasma treatment stimulated coriander germination only when nitrogen gas was used, but treatments in air and argon gases did not affect germination [92]. The authors found that plasma-generated NO gas also stimulated germination and concluded that NO causes this effect. In contrast, irradiation of radish seeds with DBD plasma using various feeding gases showed that the N_2_, He and Ar gases did not promote seedling growth, while plasma irradiation with air, O_2_, and NO (10%) + N_2_ improved plant growth [74]. Moreover, humid air plasma irradiation was more effective compared to dry oxygen. The authors concluded that the hydroxyl radical ^•^OH and excited oxygen O were the key species responsible for the effect of NTP treatment on the growth of radish seedlings. The effects of atmospheric pressure DBD plasma on wheat germination, seed coat surface changes and permeability were compared using O_2_, air, Ar, and N_2_ as feeding gases [99]. In this study, O_2_ plasma was not effective, but seed treatment for 4 min with air, N_2_, and Ar plasma increased the germination potential by 24, 28 and 36%, respectively. These results were supported by a later study [98], in which low-pressure DBD plasma was applied, and the stimulation of wheat germination was stronger using the mixture of Ar/air compared to Ar/O_2_ mixture. The effects of NTP on seed germination and growth of mung bean were strongly dependent on the feed gases used to generate plasma in the atmospheric-pressure microplasma array device [103]: air plasma was the most effective; O_2_ gas also significantly stimulated germination and seedling growth; seed treatment with N_2_ and He plasma did not have an impact. The effects of pea seed treatment with diffuse coplanar surface DBD working at atmospheric pressure were compared in ambient air, O_2_ and N_2_ atmosphere [72]. However, in this study, positive effects on germination were absent, and the longer duration of all treatments inhibited germination and caused genotoxic effects. In this respect, N_2_ plasma was the most effective, while the negative effects of air and O_2_ plasma were similar [117].

In summary, the results obtained by different studies on the effects of different feeding gases [74,86,92,98,99] are rather controversial. Despite numerous efforts, the dependence of the observed NTP effects on the physical, chemical and biological components of the NTP interaction with seeds is still far from being understood.

In many studies, the dependence of the exposure time on the effects of the treatment was studied. The duration of plasma treatments used by different authors varied broadly. Some studies observe effects after 0.5 s of treatment [61,87], while others apply treatments for 27 min [67] or even 130 min [62]. This may depend on both the plasma discharge parameters (used equipment) and the response of the plants. An optimal dose has been demonstrated for different plants [57,59,65,66,68,69,79,87,96,99,104,106,116]. That is, certain treatment durations stimulated seed germination, but adverse effects were observed when treatment duration exceeded the optimal value.

The dependence of seed NTP treatment effects on the germination (as well as other effects of NTP) and physiological seed status varies strongly due to a high level of complexity of the biological subject of research. The same treatment protocols can stimulate germination in some plant species but have no effect or even inhibit the germination of other plants. For example, the same duration of low-pressure CP treatment did not affect germination and early growth of blue lupine, but stimulated spring wheat and maize germination [49]; the germination of black mulberry was stimulated by 7 min treatment with a low-pressure CP device, while rhododendron germination was inhibited by the same treatment [47]. The same study [47] reported that the effects of seed treatment with NTP for black mulberry and rhododendron were stronger for freshly harvested seeds compared to the effects observed for the same seeds stored for 6 months. Similar results were obtained when treatment effects were compared in seeds of red clover cultivar ‘Vyčiai’ stored for different durations of time after harvest—the effects gradually decreased with an increase in seed storage time [54]. However, opposite results were obtained for radish seeds: NTP-induced positive changes in seed germination were stronger in seeds stored for 2 years after harvest compared to seeds harvested one year before the experiment [78].

Moreover, a significant difference in the response to seed treatment with NTP was observed in three different pine species [112] or in different cultivars of pea [37], poppy [116], industrial hemp [105], rapeseed [52,118], brown rice [79], red clover [54], barley [90], wheat [110], and buckwheat [109]. Differences in the effects on germination and growth have been reported even for different genetic families (half-sib) of Norway spruce [70]. In addition, the response to NTP treatment for the same lot of radish [78] or red clover [55] seeds was dependent on the color of the seeds. Such intraspecies differences and the dependence on multiple factors indicate that the response of plant germination and growth to seed treatment with NTP is determined by factors associated with slight genetic differences, peculiarities of seed structure, or physiological seed status.

Numerous studies [57,83,87,93,99,109] have reported that the effects of NTP treatment on germination were followed by similar effects on early seedling growth (from 4 days to 2 or 3 weeks). However, some studies showed that the effects on plant growth for longer periods do not correlate with the effects of NTP on germination. The negative or neutral effects of treatments on seed germination in vitro were followed by improved plant growth on a longer time scale for rhododendron [47], red clover [54], radish [76], common buckwheat [109], Norway spruce [113], and wheat [119]. In contrast, a strong positive effect of NTP on hemp germination was associated with a reduction in female plant growth [48]. Similarly, increased germination was followed by a lower growth rate of pea seedlings [120]. Such findings raise doubts about the validity of in vitro germination tests as a generally accepted criterion for evaluating the effects of the seed treatment.

Additional evidence supporting such doubts comes from comparing the effects obtained by in vitro germination tests with the results of seedling emergence in a substrate or in the field (i.e., under conditions more relevant for agricultural applications). For example, treatment of Norway spruce seeds with low-pressure CP for 2, 5 and 7 min had a strong inhibiting effect on germination in vitro, and this effect increased with the duration of treatment, but only a 5 min treatment inhibited seedling emergence in the substrate [113]. Treatment protocols with the strongest positive effects on *Andrographis paniculata* seed germination in vitro inhibited seedling emergence 7 days after sowing. They were ineffective 30 days after sowing, and the highest percentage of strong seedlings 30 days after sowing was registered for the treatment protocol (5950 V, 10 s) that did not induce changes in the seed vigor index determined in vitro [69]. Although in vitro sunflower germination was stimulated by seed treatment with low-pressure CP for 7 min, such treatment did not influence seedling emergence in the substrate [58]. A decrease in sunflower emergence induced by seed exposure to DBD plasma for 11 min was observed, while in vitro germination was not affected by the same treatment [81]. Treatment of industrial hemp seeds with low-pressure CP for 5 min increased the maximal germination percentage and germination rate in vitro, but germination yield in the field was reduced compared to the control [48]. The in vitro germination test did not reveal the effects of low-pressure CP for two buckwheat cultivars, but the percentage of seedlings that emerged in the field was significantly reduced [109].

The observed differences between NTP effects on in vitro germination kinetics and seedling emergence can be explained by several reasons. Germination in vitro is a measure of germination *sensu stricto* [43], while emergence becomes visible in the later stage of seedling growth. Numerous other factors can be responsible for differences in germination and seedling emergence kinetics, such as water penetration rate, supply of oxygen and light [46]. The presence of various compounds in the substrate and chemical interactions with the soil microbiota may also affect the seed germination rate in the substrate (all these factors are absent when seeds germinate in the Petri dish). However, the reported discrepancy between NTP effects obtained from laboratory germination tests and counting seedling emergence [58,69,81,109,113] contradicts the widespread opinion that the effects on germination can be considered as an informative indicator of plant response to seed treatment. Moreover, such findings demonstrate the importance of longer observations (at least for the entire vegetation season) under conditions used for agricultural plant cultivation.

## 4. The Mechanisms of NTP Effects on Seed Germination

The effect of NTP treatment on seeds results from the interaction of a physico-chemical stressor and a biological system—a small embryonic plant enclosed in a covering called the seed coat (or testa), usually with some food reserve. Both interacting sides are characterized by a high level of complexity and diversity. NTP is a complex stressor consisting of different components, including an electric discharge, electromagnetic and UV radiation, changes in pressure (in the case of low-pressure NTP) and temperature, the different and unstable composition of reactive chemical species, electrons, and photons. NTP reactor construction, geometry, energies, and treatment exposures vary greatly in different studies. On the other hand, seeds also vary in size, shape, color, external and internal structure, and water content. The structural, physiological, and biochemical properties of seeds strongly depend on the plant species. In addition, seeds can also have considerable intraspecies differences due to genetic polymorphism; in addition, seeds of the same variety and from the same lot are polymorphic by degree of dormancy or other traits [45]. For example, the ability to germinate differs within a population of seeds heterogeneous by size, shape, weight, or color [121].

**Figure 1:**
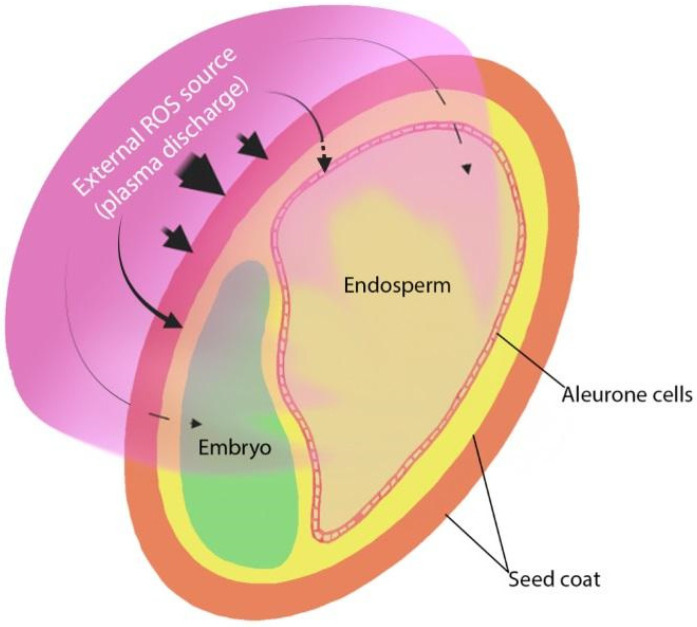
Schematic representation of NTP generated reactive species penetration into structures of the monocot seed.

Nevertheless, all seeds have three main structural parts: seed coat, embryo, and food reserve (endosperm or cotyledons) (Figure 1). The roles of these parts in the interaction with NTP may be quite different, firstly due to various locations and distances from the seed surface, i.e., the external seed layers are easily accessible for such NTP components as reactive particles, photons, and direct discharge energy, while internal structures are shielded. The biological and biochemical properties of the cells that make up these layers are also different. The seed coat is an external protective seed structure, and due to its location, plays a key role in the interaction with both physical and chemical components of NTP. Reactive species generated by plasma discharge are characterized by a short lifetime, which can be further reduced by entering the environment of reduced organic compounds within cells of a living system [122]. The distance covered in plant tissues by the most reactive species, such as the hydroxyl radical OH^•^ or peroxynitrite ONOO^−^ (half-life 10^−9^ s) is 1 nm only, for singlet oxygen ^1^O_2_ (half-life 10^−5^ s) and superoxide anion O_2_**^•^**^−^ (half-life 10^−6^ s) this distance is 30 nm [123]. Therefore, it is unlikely that these species could penetrate the seed structures deeper than the external coat surface. Other species, such as hydrogen peroxide H_2_O_2_ and nitrogen monoxide NO, are characterized by longer half-lives (10^−3^ s and 3–5 s, respectively), can easily diffuse and penetrate membranes (movement distances in order of μm) [123]. These species could be considered as candidates for reaching deeper seed layers when they originate from the outside (plasma source). However, reaching embryonal or aleurone cells for external RONS could only be possible if they are located immediately under a thin, porous, or cracked seed testa (shown by the punctured lines in Figure 1).

A model study on the penetration of NTP-generated ROS through the membrane of phospholipid vesicles (cell mimics) has been performed [124]. It was demonstrated that plasma-derived ROS are delivered into cells over a sustained period without compromising cell membrane integrity, but the presence of protein (serum) significantly reduced the transfer efficiency of ROS into the vesicles. However, the seed coat is much more complex and consists of several layers of different cells in most seeds, such as an epidermal, sub-epidermal (parenchymal) cells and a palisade layer, which often contains pigmented cells [48]. Coat cells contain numerous protective secondary metabolites and pigments that function as powerful RONS scavengers [125,126]; therefore, their amount can modulate the penetration and the effects of external RONS. Although seed cells are dehydrated [127], structures of the outer layer tissue can be strongly acidified after an interaction between external NO_2_ and NO_3_ (generated by NTP) and the remaining water. Seed exposure to external ozone O_3_ and hydrogen peroxide H_2_O_2_ also stimulates germination [128,129], indicating that the interaction of these ROS with the seed surface could contribute to the effects of NTP.

### 4.1. The Role of NTP-Induced Physical and Chemical Changes in the Seed Coat

In general, the interaction of NTP with different organic surfaces results in chemical modifications, changes in charge and structure [130,131]. The RONS and UV-photons generated by NTP react with the components of the seed surface or penetrate the external layer of the seed coat, inducing significant changes in the elemental composition of the seed coat, while prolonged treatments result in surface etching that is visible in scanning electron microscopy (SEM) pictures. One of the major chemical changes induced by NTP in seed coats of various plants is a decrease in surface carbon content accompanied by an increase in surface oxygen content (an increase in the O:C ratio), while nitrogen and silicon content remain unchanged [51,53,61,68,78,83,132,133]. Additional elements appear on the seed surface (magnesium, calcium) and other elements are found in trace amounts. Using X-ray microfluorescence, Ambrico et al. demonstrated redistribution of P, K, Mg and Zn among the different parts of basil seeds after plasma treatment [83]. Energy-dispersive X-ray analysis performed on radish seeds [78] revealed an increase in the content of C, O, Mg, Al, Si, Cl, K, and Ca after treatment; minerals such as P and S, usually located along the embryo axis [12], were detected on the seed surface after treatment. Furthermore, NTP reduced seed pH [51,134] and increased seed surface saturation with charged oxygen and nitrogen groups, as demonstrated for wheat [51], quinoa [53], beans, and lentil seeds [45]. Analysis of NTP effects by attenuated total reflectance—Fourier transform infrared spectroscopy (ATR-FTIR) was used to characterize the presence of specific chemical groups on the surface of maize [111] and pine seeds [112]. The results indicated the presence of polar nitrogen and oxygen-containing groups and the removal of lipids from the seed surface.

The introduction of polar groups and the removal of hydrophobic substances leads to chemical etching of the seed coat structure and increased hydrophilicity of the seed surface. In numerous studies carried out on seeds of different plants, seed coat erosion after exposure to NTP was observed by structural changes in SEM images, and it was evident that the degree of coat etching increased with the duration of the treatment [45,46,47,49,51,53,60,62,68,69,71,72,79,86,89,90,92,98,99,102,103,106,110,135]. In some studies, such etching was not observed, possibly due to the shorter duration of NTP treatment [78,85]. These findings led to the conclusion that the stimulating effects on seed germination depend on NTP-induced changes in the physical and chemical structure of the seed coat, and increased hydrophilicity leading to improved wettability and faster penetration of water into the seed after imbibition [45]. Changes in seed surface wettability are measured by water contact angle, and the fact that NTP treatment enhances wettability is well documented (e.g., [45,51,52,57,61,67,72,88,97,100,103,112]). Some studies have also evaluated the water penetration rate into the seed (e.g., [45,57,71,88]) and the results are fully consistent with the statement that water penetration rate is increased in seeds treated with NTP compared to the control.

The correlation of germination rate with changes in seed surface structure after plasma treatment has been demonstrated in numerous studies. However, certain inconsistencies can be observed in the reported relationships between changes in seed wettability and the effects of NTP on germination. For example, wheat germination was not stimulated by NTP despite a very strong increase in wettability [45]. Water contact angle increased with the duration of treatment with coplanar DBD system in pine seeds; however, after 60 s of treatment, a strong inhibition of germination was observed despite the maximal effect on wettability. Similar results were obtained on wheat seeds [51]. Such findings indicate that although the suggested hypothesis that NTP effects on germination can be explained by enhanced wettability and water absorption sounds rational, such an explanation is not sufficient. Water penetration is a key event to initiate germination; however, seed germination is a complex process, controlled by many other determinants besides water.

### 4.2. The Impact of Seed Dormancy and Phytohormones

Seed formation completes the process of reproduction of the plant vegetation cycle. Many seeds are dormant immediately after maturation; in this state, seeds do not germinate for some time, even when the environmental conditions are favorable for germination [136]. Dormancy is an evolutionarily developed physiological and biochemical mechanism important for plant survival as it prevents premature germination, helps seeds remain viable for a longer time in the soil and under adverse environmental conditions, and favors seed distribution over long distances.

Seed dormancy is divided into exogenous (imposed by the seed coat), endogenous (related to the embryo), and combinational dormancy (caused by exogenous and endogenous reasons) [137]. A more detailed classification system distinguishes five classes of dormancy [43,137]: (1) *physical dormancy* (denoted as PY, is determined by exogenous factors—the seed coat forms a barrier to seed germination); (2) *physiological dormancy* (PD, is an endogenous state of dormancy caused and maintained by phytohormones that inhibit germination); (3) *morphological dormancy* (MD) is also endogenous—seed embryos are not fully developed or immature, although differentiated. Such seeds require time to fully develop, sometimes 4 and more years; (4) combinational dormancy (PY + PD) is determined by internal and external factors, which are a combination of physical and physiological dormancy (germination is restricted by the seed coat and phytohormone-inhibited embryos); non-dormant seeds, ND—dormancy is naturally absent or alleviated.

Physiological dormancy is the most common type among seed-producing plants, characteristic of most (about 80%) plant species [136]. This type of dormancy is under the strict control of phytohormones, a diverse group of biologically active signal molecules comprising a complex network that controls virtually all processes in plants [43,138]. Two antagonistic phytohormones: abscisic acid (ABA) and gibberellins (GA) [43,139,140], are central to the regulation of seed dormancy and germination, but numerous other hormones are involved: auxins, cytokinins, salicylic acid (SA), and jasmonates may all be modulators of effects of ABA and GA [141,142]. In addition, brassinosteroids, ethylene, and NO are also recognized as supplementary regulators of germination [142,143,144]. ABA is a germination inhibitor, strongly suppressing the synthesis of the germination promoters, the GAs. A high concentration of ABA (and low level of GA) in dormant seeds maintains dormancy, and gradually decreases with seed storage time during natural dormancy alleviation (after-ripening) [139,140,141], i.e., the transition from a dormant to a germination competent state is associated with an increase in the GA/ABA ratio. Here, it is pertinent to mention, that the biological effects of certain phytohormones in a tightly coordinated hormonal network depend not on their absolute concentrations, but on their ratio with other hormones, since the functions of each phytohormone are modulated by other, antagonistic and synergistic hormones [145]. Seed exposure to various dormancy-breaking agents (such as stratification, scarification or chemical treatments) stimulates germination due to an increase in GA/ABA [136]. The explanations of the effects of NTP on seed germination could be related to different dormancy types, as shown in Table 2.

For example, NTP effects for plant species characterized by physical seed dormancy (e.g., legumes) can depend on NTP-induced modifications on the surface of the seed coat, leading to improved wettability and permeability to water and gases, as well as leakage of germination inhibitors from the imbibed seed. For species belonging to the physiological dormancy type, changes in the seed phytohormone balance should be more important. Stimulation of germination in seeds with an underdeveloped embryo is hardly possible, since such an embryo needs time to achieve the germination competent state. Despite several repetitive NTP treatments, our efforts to stimulate germination of morphologically dormant seeds of European ash and English yew (both belonging to MD dormancy class), were unsuccessful—seeds did not germinate either in control or treated groups (data not published). The effects on seeds of the combinational dormancy class should be determined by the changes in the seed coat and the amounts of phytohormones. Non-dormant seeds germinate rapidly without stimulation, therefore NTP treatment can be ineffective, while the effects on seedling growth are still possible. In the case of physiological dormancy, freshly harvested seeds are dormant, but their dormancy is gradually alleviated due to after-ripening (related to a progressive increase in GA/ABA, changes in gene expression, and numerous biochemical changes [146]).

The dependence of the dormancy status on seed storage time could explain the variations in the observed effects of NTP on the same seeds tested at different times after harvesting, which is observed in some studies [47,54].

Several attempts to study the NTP treatment-induced changes in the amounts of phytohormones in dry seeds have been performed using high performance liquid chromatography (HPLC) analysis [55,58,75,81,95,147,148]. Taking into account that HPLC is not sufficient for the quantitation of phytohormones, the validity of the published results must be re-evaluated using a combination of HPLC with mass spectrometry (MS), an adequate method for phytohormone analysis.

In the later study [78], LC/MS analysis was used and it was reported that the stimulation of radish seed germination after treatment with DBD plasma is related to changes in ABA and GA content. Moreover, it was demonstrated that the amounts of phytohormones involved in germination control and the shift in the GA/ABA induced by DBD plasma treatment depend on seed color. For example, in grey radish seeds (2017 harvest) DBD plasma treatment increased in GA/ABA ratio (as recalculated on pmol/g basis from data published in [78]) much stronger (5.3 times) compared to brown seeds (1.3 times). Treatment of grey (but not brown) seeds with a DBD plasma-induced positive effect on maximal germination percentage and seedling growth. The effects of seed treatment with DBD plasma in seeds (2018 harvest) on GA/ABA, germination kinetics were less pronounced in seeds of both colors.

In summary, the reported results [78] provide evidence that at least for some plant species, NTP treatment can stimulate germination due to induced changes in seed phytohormone content, that is, an increase in the GA/ABA ratio. Rapid decrease in ABA content [75] indicates that NTP is an efficient dormancy-breaking agent. This also places phytohormones in the up-stream part of the signal transduction pathway(s) that mediate the effects of NTP on plants.

### 4.3. Involvement of the Internal Generation of Reactive Oxygen Species in the Effects of NTP on Germination and Plant Growth

Plant cells possess numerous enzymatic systems for the generation of different ROS and RONS species [122,149,150,151,152]. These activities comprise an inherent part of the normal metabolism, and the presence of ROS sensing mechanisms determines the key role of ROS in the response of plants to physiological and environmental stimuli through highly complex signal transduction processes [35,151]. The production of reactive species is strongly enhanced under biotic or abiotic stress [35,122]. Both H_2_O_2_ and NO are also important signaling molecules involved in the regulation of physiological plant processes including seed germination [153,154,155,156]. The crosstalk between phytohormones, and ROS regulates seed dormancy and germination specifically [157,158]. Therefore, internal ROS and RNS generation systems should be involved in the biochemical and physiological responses of seeds exposed to NTP treatment.

ROS-induced ROS release has recently been described in plants as the mechanism that mediates long-distance rapid systemic signaling in response to biotic and abiotic stress [159]. That is defined as the production of ROS by one cell that triggers the enhanced production of ROS by a neighboring cell so that a process propagates from one part of the plant to another. This mechanism could be involved in the transfer of NTP-induced signals from the external layers of the seed coat to internal seed structures (endosperm or embryo) (Figure 1). However, it is not easy to obtain experimental evidence for such a hypothesis.

Estimation of the direct effects of NTP treatment on ROS production in intact seeds also represents a methodical challenge. Indirect evidence is provided by several studies that report an increase in the number of paramagnetic centers in seed after NTP treatments detected by EPR spectroscopy [78,113]. Significant enhancement of EPR signal (up to 30% compared with the control) was observed in Norway spruce seeds 20 h after treatment with low-pressure air NTP and radiofrequency electromagnetic field (EMF) [113]. Seed EPR spectra are composed of signals assigned to Fe(III), Mn(II) and stable organic radicals—lipid peroxides, melanin-type pigments and semiquinones originating from oxidized antioxidants located in the seed coat [160,161]. The interaction of antioxidants with reactive species generated by NTP may lead to an increase in EPR signal; however, the finding that EMF treatment also enhanced EPR signal (to a smaller extent compared to NTP) indicated a possible contribution of the internal ROS generating systems in this response of the seed to experienced stress. The results of this study were confirmed on radish seeds, treated with DBD plasma [77]. In addition, it was demonstrated that the increase in EPR signal is higher in grey seeds compared to brown seeds [78].

In several studies, the amounts of different ROS or RONS in the control seeds and those treated with NTP were compared. Low-pressure DBD treatment significantly (up to 3 times) increased H_2_O_2_ concentration in wheat seeds [89]. Ar/Air plasma induced a larger increase compared to Ar/O_2_ (stimulation of germination by Ar/Air treatment was also stronger compared to Ar/O_2_). None of these treatments affected the concentration of NO in wheat seeds. However, other authors [51] did not find H_2_O_2_ in the control or atmospheric DBD plasma-treated wheat seeds, although increased amounts of nitrites and nitrates were detected in the treated seeds. An increased amount of superoxide anion and NO and the intensity of infrared absorption of the hydroxyl group were detected in *Arabidopsis thaliana* seeds treated for short duration (up to 3 min) DBD (air) plasma, related to stimulation of germination [87]. Longer treatments resulted in an increased level of H2O2 and inhibition of germination. Still, it is not clear what part of the increase of RONS amounts found in NTP treated seeds is due to the internal ROS and RONS generating systems. On the other hand, some studies have demonstrated an increase in RONS level in young seedlings growing from NTP treated seeds [72,89,98], and that provides an argument for internal generation. Modulation of H2O2 release from germinating Norway spruce seeds treated with NTP and EMF have also been demonstrated [113].

### 4.4. Effects of Plasma Treatment on Enzymatic Activities in Dry and Germinating Seeds

Germination, or the appearance of a new plant from a seed, begins with the absorption of water by the dry seed and is completed when the elongating radicle breaks through the seed coat. Hydration of a dry seed is an essential step for seed germination. In addition, numerous external and internal factors, such as seed coat structure, physiological seed condition (dormancy, senescence, etc.), temperature, availability of light, oxygen, stimulators or inhibitors of germination may exert a strong impact on germination kinetics [43]. Biochemical and physiological activities in seeds are activated within minutes of a cell becoming hydrated and oxygenated, even before seed tissues are fully imbibed. At this stage, the rates of numerous metabolic processes such as mitochondrial respiration, selective translation, and degradation of stored mRNAs, DNA repair, synthesis and translation of new mRNAs are increasing with substantial transcription of new genes. These processes lead to the mobilization of reserves required for the rapid growth of embryo cells and their division.

Processes initiated in a dry (not imbibed) seed by NTP treatment may interfere in different ways with the complex molecular machinery which is switched on in the imbibed seed. DNA methylation is a conserved epigenetic modification that is important for gene regulation, genome stability, and plant development; it is also involved in plant responses to biotic and abiotic stress conditions [161]. The dynamics of DNA methylation are significantly impacted by oxidants, such as ROS and NO [162], therefore, at least some of the NTP effects could be related to DNA methylation in dry seeds or in growing seedlings. Until now, NTP-induced epigenetic DNA modification has been reported in a single study, carried out on heat-stressed dry rice seeds [80]. DNA methylation level was modified in the promoter regions of genes encoding enzymes of ABA biosynthesis and degradation, as well as α-amylase genes. Seed treatment with DBD plasma caused significant hypermethylation of the OsNCED5 promoter and hypomethylation of the OsAmy1C and OsAmy3E promoters, and these changes matched their expression patterns. The authors concluded that NTP could facilitate germination by upregulating ABA catabolism genes and downregulating ABA biosynthesis genes in heat-stressed seeds [80]. NTP also restored the expression of α-amylase genes in heat-stressed seeds to the level of control. This enzyme is crucial for starch mobilization in the endosperm during germination [43].

NTP effects on various enzymes in affected dry seeds, resulting in facilitated mobilization of nutrients have been demonstrated in several studies [50,80,87,98] (Table 3).

An increase in the activities of hydrolytic enzymes (amylase, protease, and phytase) along with a decrease in trypsin inhibition activity and phytic acids was reported in dry seeds of mungo bean (*Vigna radiata*) treated with low-pressure plasma [50]. NTP treatment increased the expression of amylolytic enzyme pullulanase in spinach seeds [146]. In wheat seeds [98], activities of superoxide dismutase (SOD) and ascorbate peroxidase (APX) showed no significant changes in response to any of the plasma treatments compared to the non-treated seeds. However, Ar/O_2_ (but not Ar/Air) treatment caused a significant increase in the catalase (CAT) activity in the seeds compared to the controls. The effects of DBD plasma on the activities of antioxidant enzymes SOD, CAT and peroxidase (POD) were determined in *Arabidopsis thaliana* seeds and in 7-day-old seedlings [87]. NTP did not affect SOD activity, strongly increased POD and decreased CAT activity in seeds, while the effects observed in seedlings were different: optimal treatment protocols enhanced the activities of all three enzymes.

## 5. Effects of Plasma Treatment on Biochemical and Physiological Processes in Growing Seedlings and Plants

A large number of studies on the effects induced by seed treatment with NTP on biochemical and physiological processes were carried out on growing seedlings or plants (summarized in Table 4). The body of the reported findings indicates multiple effects of a relatively short seed treatment with NTP on plant traits, detectable at the epigenomic, transcriptomic, proteomic, and metabolic levels and resulting in changes in numerous physiological plant processes.

### 5.1. Impact on Plant Epigenetics and Protein Expression

Epigenetic changes observed at certain regulatory sites indicate the impact of NTP-induced stress on genome functioning in growing plants. Changes in DNA methylation in the sequences of numerous genes and the up-regulated expression of mRNA of their protein products were reported in 6-day-old soybean sprouts growing from argon DBD plasma treated seeds [96]. The demethylation of cytosine was demonstrated in the regions of five genes—two subunits of ATP synthase, *ATP a1*, *ATP b1*, and *TOR*, *GRF 5*, and *GRF 6* genes. The authors explain the positive effects of NTP on seedling growth by an increase in the expression of proteins involved in stress response: enzymes important for energy metabolism, antioxidant defense, as well as important regulatory proteins, such as TOR kinase and six GRF proteins.

Several studies using a targeted gene expression analysis provided further insights into plant response to seed treatment with NTP. An experimental study performed on wheat [174] reported that seed treatment with DBD plasma resulted in increased transcription of heat shock factor A4A, which is involved in the plant response to abiotic stressors [200], as well as in an increased expression of POD and phenylalanine ammonia lyase (PAL), a key enzyme for phenylpropanoid biosynthesis [201]. Plasma irradiation upregulated transcription rates of WRKY1 transcription factor were reported for seedlings of industrial hemp [167] and blue sage [166]. WRKY transcription factors have diverse biological functions in plants, but are key players in the plant response to biotic and abiotic stresses [202]. Ghaemi et al. [166] also detected activation of *AREB1*, another transcription factor that regulates ABA signaling involved in stress tolerance [203]. Seed treatment with DBD plasma up-regulated the expression of the *LEA1 and SnRK2 genes* involved in the resistance to drought stress in wheat seedlings [81].

Li et al. [171] showed that NTP treatment of tomato seeds up-regulated transcription of *9-cis-epoxycarotenoid dioxygenase 1* (*NCED1*) that conferred ABA accumulation in a *respiratory burst oxidase homologue 1* (*RBOH1*)-dependent manner, leading to an improved tolerance to cold stress in tomato plants. In addition, a higher accumulation of transcripts from ABA signaling pathway genes was observed in 2-day-old *Arabidopsis* seedlings germinated from NTP-treated seeds, although their transcripts were significantly down-regulated after 4 days [163]. It has been proposed that NTP treatment accelerates ABA accumulation in the early growth stages and ABA regulates ROS and Ca^2+^ concentrations to affect the stomatal aperture, which is associated with NTP-induced stimulation of seedling growth. Holubova et al. [168] proposed that the accumulation of the heat shock protein HSP101 and HSP70 encoding gene transcripts is stimulated at the early stage (24 h) of maize seed germination due to the increased demand for the chaperones required to recover the cell proteins damaged by the NTP-treatment. Up-regulation of enzymes involved in the regulation of the cell redox balance, such as catalase (CAT) and superoxide dismutase (SOD), was observed in the roots of wheat seedlings [175] or the leaves of sunflower seedlings grown from NTP-treated seeds [169]. It was suggested that NTP treatment-induced upregulation of ATP synthase plays a stress-mitigating role in soybean and sunflower seedlings [96,169].

Comparative transcriptome analysis of NTP-enhanced early seedling growth in *Arabidopsis thaliana* revealed a differential expression of 218 genes mainly related to pathogen defense or stimulus/stress-response biological processes and involved genes of the MAPK signal transduction pathway or the glutathione, phytohormone or amino acid biosynthesis pathways [164]. Transcriptome analysis of sunflower seedlings revealed the effect of NPT seed treatment on the expression of genes involved in plant growth and development processes such as starch and sucrose metabolism, pentose and glucuronate interconversions, DNA replication, and plant hormone signal transduction [169].

In addition to the stress response signaling pathways, seed treatment has been shown to activate genes involved in the regulation of seedling development. Perez-Piza et al. [94] showed that the accumulation of the expansin gene (*GmEXP1*) transcript involved in root elongation was significantly enhanced in 5-days-old soybean seedlings grown from NTP-treated seeds. The positive effect of NTP on growth was associated with the accumulation of proteins involved in the regulation of cell growth and division, such as growth-regulating factor (*GRF*) and serine/threonine protein kinase *TOR* was observed in the leaves of sunflower seedlings [169]. In the related field of plasma application, an increase in root hair density was associated with PAW-enhanced growth of *Arabidopsis* seedlings, and the relationship of this phenomenon with the function of *COBRA-like 9* involved in root hair development and the cell wall modification enzymes xyloglucan endotransglycosylases/hydrolases *XTH9* and *XTH17* was confirmed by gene expression analysis [204].

Furthermore, several examples of enhanced activity of the plant secondary metabolite synthesis pathway have been described, including up-regulation of four enzymes of the cannabinoid pathway in hemp [167], deacetylvindoline-4-O-acetyltransferase implicated in alkaloid synthesis in pink periwinkle [205] or cinnamoyl-CoA reductases involved in lignin biosynthesis, as well as rosmarinic acid synthase in blue sage [166], and two key enzymes of the carotenoid biosynthesis pathway, phytoene-synthase and phytoene desaturase, in bitter melon (*Momordica charantia)* [165].

Enhanced gene expression of numerous enzymes in tomato seedlings was found after seed treatment with NTP [172]: antioxidant enzymes (POD, CAT, SOD, polyphenol oxidase (PPO), and glutathione transferase (GST)), biosynthetic enzymes (PAL and P450 family enzyme allene oxidase, 12-oxo-phytodienoic acid reductase) and enzymes involved in histone modifications (histone acetyltransferase and histone-lysine N-methyltransferases), enzymes of oxidative signaling (mitogen-activated protein kinase (MAPK) and respiratory burst oxidase (RBOH)).

The results of these studies indicate that plants respond to seed NTP irradiation by a wide scale modulation on the level of genome expression leading to multiple changes in metabolic and physiological processes. Methods that unveil the global balance of gene expression or the accumulation of proteins and metabolites of the cell can provide a comprehensive picture of the biological pathways or processes involved in the intricate response to NTP in a variety of organisms. Proteomics has been used to investigate a complex response of epithelial cells to NTP treatment, to address the perspectives of its medical application in the treatment of skin cancer [206,207].

In plants, an initial proteomics study on common sunflower (*Helianthus annuus*) response to seed treatment with a low-pressure NTP device revealed consistent but low amplitude differences in protein abundance in two-week-old shoots which implies that seed treatment did not trigger a distinct defense response or another stress-induced developmental program, but rather predetermined a subtle modulation of plant metabolic processes associated with enhanced growth [58]. Low-amplitude gene expression changes are characteristic of low-intensity stress (eustress) stimuli such as those described for low-intensity UV-B treatment [208]. Differences in protein abundance in the sunflower were mainly localized to chloroplasts of shoot tissues and were linked to regulation of the photosynthetic activity with no detectable changes in protein abundance in the roots [58]. Furthermore, the effect exerted by the NTP treatment was very similar to that of the EMF treatment leading to the conclusion that the EMF constituent of plasma was likely the cause of the observed plant response. A later study using an ambient atmosphere DBD device revealed similar low-amplitude protein abundance changes in sunflower seedlings but remarkably were mainly localized to the roots [170]. The differentially expressed proteins were involved in amino acid biosynthesis and derived compounds, lipid biosynthesis and protein metabolism, including stress response-related proteins, as well as carbon fixation and energy metabolism. It has been proposed that the discrepancies between the effects of the two plasma types could arise from the presence of atmospheric pressure air in the DBD type device that contributes to an abundance of reactive species during seed treatment, which could be implicated in the direct modulation of root metabolic and stress response processes, as well as induce changes in plant-associated microbiome [170].

It has been presumed that NTP-induced priming of seeds resulting in a long-term effect of stress or disease resistance could be mediated by an epigenetic mechanism of gene expression regulation [176,177]. In addition, this seems a plausible mechanism for the multigenerational effect on plant growth as was reported for *Arabidopsis* and zinnia [84], where the NTP treatment of seeds for two generations resulted in the most prominent growth enhancement of the plants. Recently, it has been suggested [209] that NTP-generated nitric oxide (NO) is involved in the regulation of epigenetic modifications leading to an enhanced proliferation of mammalian stem cells. NO is a well-established epigenetic modifier implicated in the regulation of gene expression and the development of animal [210] and plant [211] cells; however, its role in the NTP-induced plant response remains to be elucidated. Seed treatment performed in the argon atmosphere [96] induced DNA demethylation, therefore a direct role of NTP-produced ROS or RNS in the regulation of DNA methylation should be excluded and suggest the presence of a different mechanism for the NTP-induced epigenetic changes in seeds or implies that the changes occurred at later stages of the seed germination and seedling development.

### 5.2. Changes in Enzyme Activities

Positive effects of NTP on germination were associated with an enhanced early growth of the seedlings, and numerous reports are available on changes in the amounts of soluble protein [72,96], sugars [81,94,178], proline [178,192], ATP amount [96] or activities of different enzymes, mostly involved in the antioxidant defense, such as SOD, CAT, POD, APX, dehydroascorbate reductase (DHAR), glutathione reductase (GR) (e.g., [18,60,69,87,89,96,98,173,176,179,181,182,183,184,185,186,189]). In most of these studies, increased activities of antioxidant enzymes in seedlings growing from NTP-treated seeds were observed. These findings are in line with the accepted standpoint that after exposure to biotic and abiotic stressors plants respond to an increase in intracellular ROS levels by raising the level of endogenous antioxidant defense [212]. Thus, increased ROS amounts in NTP treated dry or germinating seeds (Table 3) are followed by an increased expression or activity of enzymes of antioxidant systems in leaves and roots of growing seedlings. An analysis on the extent of enhancement in the activity of enzymes based on different reports was provided in a recent review [17]; the results showed that even a low power and short exposure time NTP treatments can strongly induce cellular antioxidant systems. The largest increase (up to 100% compared to the untreated control) was reported for POD activity, although the observed effects for POD, CAT and SOD remarkably varied. In line with enhanced expression (Section 5.1) and activities of antioxidant enzymes, decreased levels of a lipid peroxidation marker malondialdehyde (MDA) were detected in seedlings of different plants growing from NTP-treated seeds [52,69,89,186,213].

In addition, rice seed treatment with NTP also stimulated enzymes involved in the biosynthesis of secondary metabolites (SMs), such as PAL, polyphenol oxidase (PPO), shikimate dehydrogenase (SKDH), cinnamyl alcohol dehydrogenase (CAD), as well as enzymes of the primary metabolism (sucrose synthase, sucrose phosphate synthase, and acid invertase) under normal and salinity stress conditions [182].

### 5.3. Effects on the Amount of Phytohormones in Plant Tissues

Phytohormones actively interfere with different signaling pathways, therefore, changes in their amount and balance may be responsible for the multifaceted response to NTP treatments triggered in plants. The expression of genes responsible for the synthesis and degradation of phytohormones and the amounts of phytohormones in seeds is strongly affected by NTP treatments (Table 2). The existing data also reveal significant changes in the amounts of phytohormones in seedlings growing from the treated seeds [108]. It has recently been reported that the positive effects of gliding arc plasma treatment on maize seed germination and seedling growth were related to significant changes in the amounts of phytohormones in the leaves of 14-days old seedlings [108]. The effect of NTP on phytohormones was dependent on the duration of the treatment: short-term (180 s) plasma treatment decreased the levels of stress hormones (ABA, SA, JA and JA isoleucine) as well as active cytokinins, while longer exposures (300 s, 600 s) that had a stronger effect on plant growth, led to their increase. The elevated amount of cytokinins after longer exposure correlated well with an enhanced germination and seedling growth.

The effects of seed exposure to DBD plasma on the content of auxins and cytokinins in 14- and 21-days-old pea seedlings were studied by Stolaric et al. [71]. The results indicated that NTP treatment increased the biosynthesis of some auxins and cytokinins, as well as their catabolites and conjugates. The authors concluded that such changes can lead to improved seedling growth. Tomato seed treatment with NTP resulted in enhanced levels of three cytokinins and reduced ethylene concentrations in leaves exposed to darkness [173].

Reports have been published on NTP treatment-induced changes in amounts of phytohormones in wheat [89] and soybean [95] seedlings. However, only HPLC did not allow for analysis in these studies. The quality of the performed HPLC detection [89,95] was not sufficient for quantitation of phytohormones, in contrast to the LC-MS/MS analysis with internal standards as performed in [71].

### 5.4. Effect on Photosynthesis

NTP-induced stimulation of plant growth can be explained by stimulated photosynthesis. Numerous studies reported an increase in the amount of photosynthetic pigments in the leaves of seedlings growing from NTP-treated seeds. Such results were obtained for carrot, wheat, maize, rice, spinach, tomato and other plants (Table 4). Thus, positive NTP effects on photosynthesis may be at least in part related to an enhanced content of chlorophyll, which is an important component of the plant photosynthetic system. NTP-induced changes in the expression of numerous proteoforms associated with photosynthetic machinery in sunflower seedlings [58] can be associated with improved photosynthetic indices [81]. Positive effects on the parameters of photosynthetic efficiency were also observed in growing plants of common buckwheat [109], soybean [189], and purple coneflower [190]. Increased activity of photosynthesis in seedlings growing from low-pressure CP treated seeds was observed in wheat [191]. The same was found for DBD plasma on maize [179], while seed exposure to gliding arc plasma did not induce activation of photosynthesis in maize seedlings [108]. In pea seedlings, a decrease in the efficiency of photosynthesis along with retarded growth was reported [120], in line with the statement about a close relationship between the photosynthetic function and plant growth.

### 5.5. Changes in Amount of Secondary Metabolites

Plants synthesize countless secondary metabolites (SM) of diverse chemical structures and biological activities. SMs, such as terpenes, phenolics, polyketides, and alkaloids, play an important role in plant defense and adaptability to the changing environment [214]. In plants, SM function as signaling compounds, antibiotics, antioxidants, allelochemicals, chelators, pheromones, toxins, differentiation effectors, communication means, etc. In addition, most of the SMs are biologically active substances exerting various effects on the cellular processes in other organisms, therefore SMs are responsible for the medicinal value of plants and have a wide range of practical applications, particularly in pharmaceutics and food industry (as food additives) [215]. It is well established that plant response to biotic and abiotic stressors involves an increased accumulation of SMs [216,217]. Among the many molecular signals involved in the enhancement of SM synthesis in response stress, central roles belong to the phytohormones SA and JA [214], therefore, NTP-induced changes in SMs amounts described below may be caused by NTP-induced changes in the amounts of phytohormones. However, an increase in plant SMs induced by stress experienced in a seed stage was not highlighted. An increase in the amounts of SM in plants growing from NTP treated seeds was documented in numerous studies (Table 4).

An increase in phenolic compounds after seed treatment with NTP was observed for wheat, brown rice, buckwheat, spinach, purple coneflower, red clover, maize, Norway spruce, and coriander (Table 4). In the study of Yodpitak et al. [79] on the six cultivars of brown rice, the concentration of secondary metabolites was periodically measured in extracts of germinating seeds. The maximal increase in concentrations of phenolic compounds, anthocyanins, phytosterols, triterpenoids, and vitamin E was reached one day earlier after NTP treatment compared to the control groups. Low-pressure oxygen CP modulated the amount of glucosinolates in rapeseed seeds, and the effects differed among cultivars: a decrease was found in ‘Westar’ and ‘Kizakinonatane’, while an increase was detected in ‘Nanashikibu’ cultivar [118]. Such findings [79,118] imply that NTP effects on SM synthesis NTP are observed even in germinating seeds.

Treatment of purple coneflower seeds with low-pressure CP for 2, 5 and 7 min resulted in a drastic increase in the content of phenolic acids, vitamin C and radical scavenging activity in the leaves of a widely used medicinal plant [190]. Such NTP effects (increasing biomass, content of biologically active SMs, antioxidant activity) may be relevant to increasing the production of natural products. In the leaves and roots of red clover, low-pressure CP also induced substantial changes in the content of isoflavones. The total amount of the two major isoflavonoids was increased only in ‘Sadūnai’ cultivar, but in both ‘Sadūnai’ and ‘Vyčiai’ cultivars, NTP induced remarkable changes in the ratio of formononetin and biochanin A [54]. The composition of flavonoids in root exudates of red clover plants growing from NTP treated seeds also significantly differed from the control plants, so that the amount of 7,4′-dihydroxyflavone, daidzein, quercetin, and kaempferol was increased more than twice [148]. A strong increase in daidzein and formononetin content in the roots of red clover plants grown from low-pressure CP treated seeds was reported by other authors [193], although isoflavonoid changes in the leaves were different from those obtained in another study [54]. The amounts of isoflavonoids daidzein, genistein, and daidzin were diminished 1.5–1.8 fold compared to the control (while genistin content was not affected) in the roots of soybean plants growing from DBD plasma treated seeds [94]. DBD plasma-induced changes in the amounts of photosynthetic pigments and the total phenolic compounds were different in seven genetic families of the Norway spruce [70].

The amounts of quercetin glycosides and kaempherol glycosides increased in pea seedlings growing from DBD plasma treated seeds [120]. Treatment of industrial hemp seeds with low-pressure CP reduced female plant growth and the amount of cannabidiolic acid (CBDA) in inflorescences, while vacuum treatment significantly increased it [48]. An increase in the amounts of anthocyanins after NTP treatment was reported in the roots of maize seedlings [192] and in wheat leaves [191]. Certain NTP treatment-induced changes were found even in the harvested seeds of common buckwheat: the content of rutin was not affected, but the amount of quercetin was higher in the seeds of ‘VB Vokiai’ and ‘VB Nojai’ cultivars, although longer treatment (for 7 min) reduced it in ‘VB Nojai’ seeds [109].

Thus, numerous studies provide evidence that changes in SM content of seedlings are characteristic of the response of different plant species to seed treatment with NTP (Table 4). Along with the enhanced expression and activities of antioxidant enzymes [18,60,69,87,89,96,98,173,183,184,185,186,189], an increased level of SMs may be important for the improvement of plant fitness, adaptability and disease resistance.

### 5.6. NTP Effects on Plant Adaptability and Stress Resistance

Plants, as sessile organisms, are tied to their habitat and require efficient strategies to avoid or adapt to stress [214]. Depending on nature, plant stressors can be divided into abiotic (drought, heat, salinity, high light, mineral deficiency, low temperature, wounding, ozone, UV-A, UV-B), biotic (insects, pathogens, elicitors, bacteria, fungi, virus), and anthropogenic (herbicides, air pollution, peroxyacyl nitrates, radicals, acid rain, acid fog, heavy metal load) [214,215,216,217,218,219,220,221] With the growing anthropogenic stress load, climate change, and human population growth, innovative ways for making crops more resilient to environmental stressors are highly demanded. To date, several studies have demonstrated that seed treatment can induce adaptive plant responses to various environmental stressors.

Evidence for NTP effects on plant adaptability to abiotic stress (such as chilling, draught, salinity) have been obtained in several studies [52,67,80,89,165,172,174,178,182,222]. NTP-treated cotton seeds were subjected to germination tests that can indicate the ability of the seed to overcome adverse environmental conditions, and the results revealed that NTP treatments enhance germination rates under warm-germination or metabolic-chilling conditions [67]. Wu et al. [178] observed the improved resistance of maize to salt, cold and drought stress at the seedling stage after NTP treatment. Li et al. demonstrated that NTP treatment stimulated oilseed rape seed germination and improved morphometric parameters of seedlings under drought conditions for drought-sensitive and non-sensitive rape cultivars [52]. Sheteiwy et al. [182] found that rice seed treatment with NTP stimulated seedling growth, enhanced the activities of antioxidant enzymes, as well as enzymes involved in SM biosynthesis and primary metabolism, improved the uptake of macro- and micronutrients, resulted in a significant decrease in ROS and MDA contents and helped the plants to recover their cell turgidity under salinity stress. Improved resistance to salinity stress was found in wheat seedlings growing from DBD plasma-treated seeds [174]. Such an effect of NTP was associated with an up-regulated expression of heat shock factor HSFA4A in the roots and increased activities of PAL and POD. The effect of low-pressure CP (air and helium) treatments on the germination and seedling growth of alfalfa seedlings under simulated drought stress conditions was investigated [222]. The authors concluded that NTP treatment had a significant effect on the adaptability of alfalfa seeds in different drought environments since vigor indexes of the treated seeds under different extents of drought stresses were higher compared to untreated controls. Alleviation of the adverse effects of drought stress on wheat germination and seedling growth was induced by DBD treatment, and it was associated with an enhanced ABA synthesis and SOD, CAT, and POD activities, increased amount of proline and reduced ROS content under drought stress [89]. Similar changes along with enhanced tolerance to drought stress was observed in tomato seedlings growing from plasma jet treated seeds [172]. It was found that that treatment of rice seeds with DBD plasma treatment significantly improved the germination of seeds exposed to high-temperature stress during grain filling [80] and these effects are related to changes in epigenetic regulation and expression of genes involved in ABA synthesis and degradation as well as several α-amylase genes.

Several studies have reported an improvement in the adaptive plant response to anthropogenic factors after NTP seeds treatment, such as contamination with toxic chemicals, heavy metals, and nanoparticles. Pre-sowing seed treatment with NTP reduced DNA damage in pea seedlings caused by a toxic concentration of radiomimetic zeocin [223]. Similarly, priming of seeds with NTP seed activated defense-related mechanisms and mitigated toxicity signs of selenium and zinc oxide nanoparticles for lemon balm and bell pepper plants, improving their growth-related characteristics [177,181], similar effects were observed for *Astragalus fridae* seedlings grown in an in vitro culture medium supplemented with silicon nanoparticles [176].

An improved adaptability of plants to biotic stressors was demonstrated by NTP-induced effects on plant resistance to pathogens. It was found that DBD plasma treatment applied to soybean seeds with a high incidence of seed-borne pathogens (*Diaporthe*/*Phomopsis* complex) increased plant growth and alleviated the negative effects of the disease in seedlings (reduced photosynthetic performance, chlorophyll content, discoloration, retarded growth) [189]. The authors came to the conclusion that the effects of NTP treatments are partially dependent on the removal of pathogens from seeds, however, the impact of NT—induced changes in seedling antioxidant defense potential and content of SM also cannot be excluded.

The effect of pre-sowing treatment of maize, narrow-leaved lupine and winter wheat seeds with low-pressure CP on plant resistance to common diseases during vegetation and crop yield was studied in laboratory and field experiments [192]. Seed treatment suppressed a number of fungal crop diseases such as boil smut in maize, root rot in lupine and winter wheat at different growth stages. The results can be explained by both the decreased level of seed infection and changes in the defensive potential of growing plants, at least in the roots of maize seedlings NTP-induced increase in the content of non-enzymatic antioxidants (proline, anthocyanins as well as total phenolic content) was determined.

Low-pressure CP treatment increased the resistance of tomatoes to bacterial wilt, caused by *Ralstonia solanacearum* with an efficacy of 25% [224]. Such an effect was related to increased production of H_2_O_2_ and POD, PPO and PAL. NTP treatment increased both germination and plant growth, absorption of calcium and boron compared with the controls.

Thus, improved plant performance and NTP-decreased frequency of infections can be explained by both seed decontamination and mobilization of plant defense mechanisms. Changes in plant communication with beneficial microorganisms also can be involved. In any way, improved adaptability and stress can result in the better establishment of young seedlings, increased growth and yields of plant production.

### 5.7. Effects on Plant Growth and Productivity

Studies aimed to determine the effects of seed treatment with NTP on plant growth and production yields were performed on annual plants and perennials, and significant positive effects were obtained for *Arabidopsis* [85], black mulberry [47], common buckwheat [109], garlic [195], industrial hemp [48], maize [179,196,197], Norway spruce [113], purple coneflower [190], peanut [198], red clover [54], rhododendron [47], tomato [40,171,199], wheat [63,191,192,197] (Table 4).

The effects of air DBD plasma irradiation of *Arabidopsis thaliana* seeds on plant growth were studied from the beginning of cultivation to the harvest [85], and growth acceleration in all the growth stages were observed. NTP treatment for 3 min resulted in a shorter harvest period (11%), a significant increase in the total seed weight (56%), one seed weight (12%), and seed number (39%). Low-pressure CP treatment of garlic cloves induced increases in the water uptake and accelerated root growth in a laboratory experiment. The effects were not so obvious in a field experiment, although some trend for increased plant height and dried bulb mass was observed [195]. Compared to the control, plant height, root length and fruit yield (up to 26%) were enhanced and the incidence of disease was decreased in tomato plants growing from DBD plasma treated seeds [199]. Similar effects of NTP treatment on the growth of tomato plants and fruit yield were obtained by Meiqiang et al. [40]. Treatment of tomato seeds with plasma jet discharge resulted in higher shoot length (up to 36%), root length (up to 13%), fresh weight (up to 30%), and increased shoot to root ratio (up to 19%) with respect to control [171]. Positive effects of red clover seed treatment with low-pressure CP on plant biomass gain in the field 5 months after sowing were much stronger (up to 49%) in comparison to the effects (neutral or below 10%) observed in the early growth stages [54]. The field experiment that lasted 1.5-years demonstrated that low-pressure CP treatment markedly stimulated peanut germination and growth, increased branch number per plant, pod number per plant, compared to the control. The yield was improved by 10% [198].

An NTP-induced increase in wheat growth and yield was reported in several field studies. Seed treatment with low-pressure helium CP increased morphometric plant parameters (plant height, root length and fresh weight, leaf area, etc.) at seedling and booting stages, and the yield of treated wheat was increased by 6% compared to the control [63]. Glow discharge (air and air/O_2_) plasma treatment stimulated wheat germination, growth in the field and increased yield by 20% over control [197]. A field experiment on wheat seedlings growing from low-pressure CP treated seeds was repeated for two years [191], and the results showed that NTP increased biomass yield (up to 44%), grain yield (up to 35%) and 1000 grain weight (up to 22%).

Glow discharge (Ar + O2) plasma treatment enhanced the germination of maize seeds, plant growth and development, productivity (1.3%) and improved nutritional composition (moisture, ash, fat, and crude fiber) of leaves, and increased iron and zinc content in grains [180]. Cianioti et al. [196] examined the impact of maize seed treatment with DBD plasma on the germination, physiology, yield and quality characteristics of two maize hybrids with high and low germination capability. It was found that NTP significantly improved the germination and growth of both cultivars. Maize yield increased by 18–25% compared to untreated groups. Low-pressure CP treatment decreased the level of seed infection, stimulated field germination, plant growth and resistance to pathogens during the vegetation period, as a result, the grain yield increased for winter wheat and maize by 2% and for narrow-leaved lupine by 27% compared to control plants. However, in the field study performed in the field by Ahn et al., positive effects of NTP treatment on maize growth were not found [115]. In this study, corn seeds were treated by three types of NTP devices: RF plasma in a vacuum, microwave-driven atmospheric-pressure plasma, DBD atmospheric-pressure plasma, and the effects on the yield of harvested corn observed in six different locations were not significant.

In several cases, NTP effects on plant growth for longer time periods did not coincide with the effects observed on germination in vitro. Treatment of common buckwheat seeds with low-pressure CP did not affect germination in vitro and decreased the percentage of seedlings that emerged under field conditions, NTP treatment strongly improved buckwheat growth and yield, so that the weight of seeds collected per plant for both cultivars was significantly higher (up to 70–97%) compared to the control [109]. Low-pressure CP treatment inhibited germination of the Norway spruce but stimulated plant growth: 17 months after sowing, seedling height was 50–60%, the number of branches was 40–50%, exceeding the same parameters of the control plants. Similar results were obtained for rhododendron—low-pressure CP treatment characterized as distressful based on changes in germination and increased growth of seedlings (stem and root branching, leaf count and surface area) after 13 months [47].

The results of the studies described in this section lead to the conclusion that NTP effects on annual plants persist for the entire vegetation season, and for perennials—at least for several vegetation seasons [47,113,190]. In addition, seed treatment can lead to a significant increase in biomass production and grain yield. Therefore, the application potential of NTP treatments in agriculture is not limited to the effects on germination.

## 6. NTP-Induced Changes in the Seed Microbiome and Plant-Microbial Interactions

The NTP-generated reactive chemical species and UV can damage microorganisms and the technology finds versatile application as a sterilizing agent used in medical practices and the food processing industry (reviewed by [225,226,227,228,229]). The NTP treatment can be easily applied to most plant seeds or grains due to their small size and low water content. The impact of NTP on the microorganisms residing on a surface or inside seeds can have two-sided implications, which can lead to different applications. On the one hand, the antimicrobial effect of NTP prolongs the shelf life of seeds, it is beneficial for the safety of seed-derived foods, such as sprouts [230,231,232], and decontamination with NTP could reduce the occurrence of seed-born fungal or bacterial diseases [29,213,230,233,234,235]. On the other hand, the seeds carry an assembly of microorganisms that are important for the survival and vigor of the germinated seedlings and plants [236,237,238] and the NTP-mediated inactivation (or activation as suggested by [239]) of this part of the seed microbiome could lead to a long-term effect on plant development, resistance to pathogens and productivity.

Efficient microbial inactivation of seeds by NTP treatment was reported for chickpea [240], alfalfa, onion, radish, cress [241], cucumber, pepper [68], lentil [232], rice [230], buckwheat [114], barley [51] and wheat [235] grains. Incidence of fungal pathogenic strains of *Aspergillus* spp. and *Penicillum* spp. [242], *Rhizoctonia solani* [233], and bacterial pathogen *Xanthomonas campestris* [234] on seeds was largely reduced by NTP treatment in air, sulfur hexafluoride or argon atmosphere. However, filamentous fungi of the genera *Alternaria* and *Epicoccum* proved to be resistant to NTP treatment [114].

NTP treatment was also effective against bacterial spores [243,244,245]. Efficient inactivation of an indicator strain of spore-forming *Bacillus atrophaeus* was achieved by direct application of NTP [246]. However, *B. atrophaeus* or *Geobacillus stearothermophilus* endospore inactivation on barley and wheat grains was less efficient and required extended treatment [51,247]. This is presumed to result from microorganisms being sheltered by the uneven surface of grains [246,247], and the inactivation efficiency depends on the substrate moisture level and the NTP supply settings that determine the outcome of the reactive species [241].

NTP mediated inactivation of microorganism cells was linked to impairment of cell membrane and wall integrity and damage of integrity and function of intracellular components such as DNA and protein by NTP-generated ROS and RNS produced in air atmosphere [228,248]. When the treatment is carried out in an inert gas atmosphere such as argon, the sterilization effect is proposed to be related to the impact on the microbial membrane by energetic species of electronically excited inert gas ions, metastable particles and atoms [249]. In addition, a negative effect of the NTP-generated UV irradiation on microorganisms has been suggested [249,250,251,252] and discussed by [226].

Notably, the NTP effect depends on the dose and composition of generated reactive species, and plasma treatment may enhance the vitality of bacteria and their plant growth-promoting properties [239]. The microbial cell response to sub-lethal NTP doses was addressed in bacteria using proteomic and transcriptomic studies. The response of *Salmonella enteritidis* was associated with an increase in the abundance of proteins related to carbohydrate and nucleotide metabolism, suggesting an enhancement of energy metabolism [253]. Yau et al. [254] linked upregulation of bacterioferritin B protein to NTP-induced oxidative stress response in *Pseudomonas aeruginosa*. Similar activation of the oxidative stress response and DNA repair processes attributed to the concerted action of ROS and UV irradiation were revealed by gene expression analysis in *E. coli* [255] and *Deinococcus radiodurans* [256]. Argon plasma upregulated numerous genes associated with cell wall synthesis and degradation in *E. coli* cells and the response was different from air atmosphere plasma [257,258]. Krewing et al. [259] performed a genome-wide screening in *E. coli* for plasma-protective genes that confer plasma resistance. The study revealed 87 genes, most of which protect against H_2_O_2_, O_2_^−^ and NO. Upon exposure to low-temperature nitrogen gas plasma of *Bacillus cereus* cells, the transcriptome profile showed a large overlap with profiles obtained from conditions generating reactive oxygen species [260].

Plant-associated microbiota has an immense effect on agro-ecosystem health by supplying nutrients to plants or priming resistance to systemic disease [261]. A reduced microbial diversity is associated with an impairment of the normal state of a plant, often caused by pathogens [262,263]. Therefore, it seems reasonable to investigate the extent of the NTP-induced changes in the composition of microbiota vertically transmitted through seeds and their consequences on plant growth and adaptability. As has been observed for NTP-induced responses in living organisms, NTP antimicrobial properties vary depending on treatment conditions. For instance, Los et al. [51] observed no effect on overall counts of natural microbiota of barley grains upon the NTP treatment that was effective for inactivating bacterial inoculum on the seed surface. However, a significant reduction of the natural seed surface microbiota was detected by Mitra et al. [240]. Recent 16S rRNA gene sequencing-based analysis of the bacterial composition of in vitro germinated *Arabidopsis* seedlings induced by seed treatment with NTP showed a ~4-fold reduction in the number of identified bacterial genera [194]. Furthermore, the analysis of the leaf microbiome of plants germinated from the NTP-treated seeds and grown under greenhouse conditions revealed the effect of NTP on bacterial diversity [194]. A similar effect was observed in 2-week-old seedlings of common sunflower [170], and links between the changes in microbial composition and observed stimulation of root and lateral organ growth were proposed.

However, it remains to be answered whether NTP-induced changes in plant-associated microbiome occurred due to a direct effect of plasma on microorganisms residing on seed surface or inside the seeds or is a consequence of NTP-induced changes in plant and, especially, in root physiology that result in altered interaction with the soil microbiome and colonization by endophytic bacteria. An example of such interaction was obtained in a study of soybean plants by Perez-Piza et al. [94]. It was found that improvement in biometric parameters of soybean seedlings growing from DBD plasma treated seeds was associated with enhanced nodulation and stimulated N-fixation by nodular rhizobacteria. Such findings [94] could be explained by NTP-induced changes in secondary metabolism, since flavonoids released from roots to rhizosphere are recognized as signal molecules promoting the formation of nodules by symbiotic bacteria in the roots of legumes [264]. However, the content of flavonoids in the roots of soybean was decreased [94]. NTP-induced stimulation of root development and enhanced nodulation was reported in red clover roots [148], and increased amounts of flavonoids were detected in root exudates.

In any case, the performed studies [94,148,170,194] have provided evidence that NTP treatment is capable of inducing changes in the plant-associated microbiome that may mediate secondary effects on plant physiology as well as the agroecosystem environment.

## 7. Perspectives

A large number of recent studies have reported new findings based on epigenomic, transcriptomic, proteomic, and metagenomic approaches. These findings reveal the level of complexity of the molecular mechanisms involved in plant response to stress caused by short-term seed treatment with NTP. Many important aspects at the molecular level (such as DNA methylation, massive changes in gene and protein expression, the contribution of RONS and phytohormones, strong positive effects on plant growth and yield, mobilization of secondary metabolism, increased adaptability to stress, effects on the plant-associated microbiome, etc.) emerged in the last decade. However, despite invaluable progress, a complete structure of wide-scale modulations induced by interaction with NTP at the different hierarchical levels of the plant is far from being understood, and accumulative changes in the biochemical and physiological processes are under-explored.

**Figure 2:**
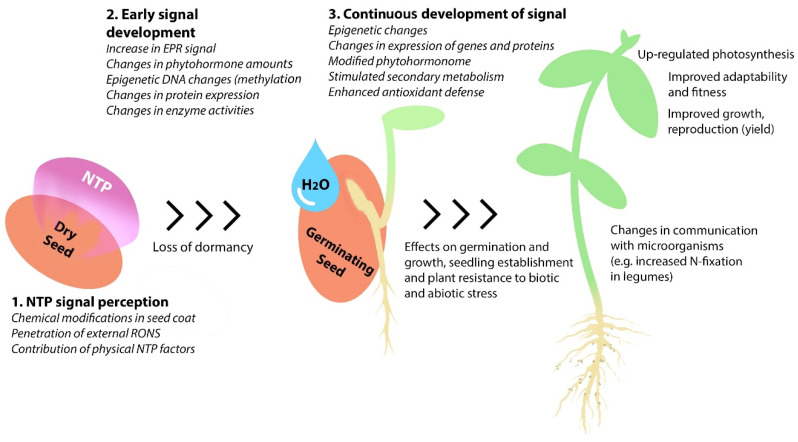
Schematic representation of three stages in the time course of the NTP-induced signal development in seeds and plants.

Taking into account that the effects of seed treatment with NTP are persistent for longer time periods (at least for the entire vegetation season for annuals, Table 4), we suggest the multifaceted effects of NTP on plants should be considered as a multi-step process (Figure 2) which starts from NTP signal perception (stage 1) and early response events in a dry seed (with few exceptions [72,167], imbibed seeds have not been exposed to NTP). Changes induced by NTP in seeds before imbibition (summarized in Table 3) comprise stage 2 of the stress response, and water penetration after seed imbibition induces the further processes (stage 3) resulting in modified kinetics of germination (Table 1) and early seedling growth (effects on seedling growth for several weeks are usually following trends of NTP-induced changes in germination). These changes have an impact on the further processes in the growing plant (at least for the entire season of vegetation in annuals).

From a research perspective, one of the most poorly defined parts of the puzzle is the molecular systems in seeds that are responsible for the perception of NTP signals. Receptors for NTP sensing in seeds have not been established; furthermore, more than one receptor can be involved in the perception of different factors constituting a complex NTP signal. The first event in the interaction of seeds with NTP is chemical and structural changes on the seed coat surface (Section 4.1), and NTP receptors are most probably located there. The possible contribution of the perturbations in membrane permeability, the activity of ion channels (e.g., Ca^2+^ channels) or ROS producing enzymes residing in the membranes of cells or cellular walls in the seed coat or in layers of seed structure underneath the coat remain to be determined. In animal cells, Ca^2+^ channels TRPA1 and TRPV1 are involved in the response to atmospheric-pressure NTP [265]. Although TRP channels are not found in plants [266], other ROS-sensitive Ca^2+^ channels function in plant cells [267]. Still, it is not clear if such channels operate in seeds. NTP-induced chemical and physical changes on the seed surface facilitate water penetration or lead to an increase in the EPR signal (Section 4.1). These effects might also be considered among the up-stream factors. ROS-induced ROS release (RIRR), a process in which one cellular compartment or organelle generates or releases ROS, triggering the enhanced production or release of ROS by another compartment or organelle, was first described in animal cells, but later it was discovered in plants [159]. It was supposed that in plants, RIRR is involved in cell-to-cell communication, i.e., enhanced production of ROS by one cell triggers the enhanced production of ROS in a neighboring cell, so that process propagates from one part of the plant to another. It is tempting to speculate about the possible contribution of the external (or NTP-generated) ROS-induced internal ROS release as one of the possible modes for NTP signal perception.

The interaction between receptors and NTP should result in the production of down-stream secondary messengers initiating the response to NTP by turning on yet unknown signal transduction pathways. Knowledge of signal perception, as well as signal transduction mechanisms, is crucial for a better understanding of the different outcomes of NTP treatments, a balance between eustress and distress response, or plant species/genotype dependent NTP effects.

A large breakthrough has been made recently in understanding signal development in stages 2 and 3. Evidence of the contribution of epigenetic DNA changes was reported and a large number of studies on changes in gene and protein expression have been published (Section 4.2 and Section 5.1). The role of NTP-induced changes in the amounts of phytohormones and RONS production in seeds has been well-documented in agreement with the basic concepts placing cross-talk between phytohormones and ROS at the core of the combinatory plant response to abiotic stress [155,214]. In this review, some rationale for a possible relationship between seed physiology (such as dormancy types) and the effects of NTP on germination was provided (Table 2). However, more detailed information on the role of miRNA, histone modifications or RONS contribution to NTP-induced changes in protein expression through the recruitment of mitogen-activated protein kinases (MAPK) and protein phophorylation, or oxidative modifications of mRNA or posttranslational modifications of proteins (such as carbonylation and nitrosylation) is still not available. The detailed structure or sequence of the involved NTP signal transduction pathways (what is up-stream, what is down-stream?) operating in seeds and in plants is not yet elucidated. Moreover, it can be expected that NTP-triggered signaling cascades are different between different plants or treatment modes.

Multiple changes started in the dry seed develop further and possibly diverge during germination and early seedling growth (Table 3). The down-stream imprint of these changes on the biochemical and physiological processes is observable for the entire vegetation season (or more seasons, for perennials) and manifests by effects on metabolic and protective enzymes, photosynthesis, secondary metabolism, composition of microbiome (Table 4). That results in improved plant growth and reproduction (seed yield), adaptability to stress, increased plant fitness, performance, and better survival chances under unfavorable conditions.

Due to their anhydrobiotic state, seeds are highly resistant to environmental factors [268]. This trait is of key importance for seed longevity, plant reproduction and survival. At the same time, the accumulated knowledge on the outcomes of NTP interaction with seeds reveals that plants have developed mechanisms to respond efficiently to short and rather moderate stress experienced at the seed (embryo) stage. These mechanisms allow seeds to sense environmental changes that could be dangerous for the survival of seedlings and enables plants to respond to such signals by mobilizing internal resources and their defensive potential, leading to improved fitness and competitiveness on a longer time scale (stimulated growth, defense and reproduction). The knowledge of such mechanisms has immense potential for applications in agriculture. However, most results on the effects of seed treatment with NTP are obtained in the laboratory or in small-scale field experiments. For the development of reliable NTP-based agro-biotechnologies, NTP treatment devices for the treatment of large quantities of seeds should be designed and NTP effects on plants should be verified in up-scaled agricultural experiments.

## Figures and Tables

**Table 2:** Possible mechanisms of NTP effects on germination for seeds with different dormancy types.

Dormancy Type	Key Determinant	NTP Effects Due to
Physical, PY	Permeability is limited by the seed coat	Changes in the surface and improved permeability of the seed coat
Physiological, PD	Phytohormone balance (low GA/ABA)	Shift in the balance of phytohormones (GA/ABA increase)
Morphological, MD Morphophysiological, MPD	Under-developed or immature embryo Under-developed embryo and phytohormones	NTP not effective
Combinational: physical and physiological, PY + PD	Germination is limited by the seed coat and inhibited by phytohormones	Combination of the involved factors (both coat and phytohormonal changes)
Non dormant seeds, ND	Seeds are germination competent	Negligible effects on germination

**Table 3:** Summary of the published findings on NTP-induced changes in biochemical processes in dry seeds.

NTP Induced Change	Plant Species [Reference]	Implication
Increased number of paramagnetic centers (EPR signal) in seeds	Norway spruce [113], radish [77,78]	Increased production of stable organic radicals indicates the interaction of seed components with ROS (NTP generated NTP or internally produced)
Increased ROS amount in dry and in germinating seeds	Wheat [89], *A. thaliana* [87], Norway spruce [113], soybean [95]	Induced internal RONS production; RONS involved in NTP effects
Change in the balance of phytohormones involved in the control of germination	Radish [78]	NTP effects on germination are related to induced shift in GA/ABA
Gene expression and expression or activities of proteins (including enzymes)	Mung bean [50], rice [56,80], *A. thaliana* [87], spinach [147], soybean [95], wheat [98]	Induced changes in the expression or activities of proteins/enzymes involved in mobilisation of resources for germination and antioxidative defense
DNA methylation	Rice [80]	NTP induces changes in gene expression through changes in DNA methylation.

**Table 4:** Summary of published findings of NTP-induced biochemical changes in growing seedlings and plants.

NTP Induced Change in	Plant Species [Reference]	Implication:
DNA methylation	Soybean [96]	Impact on gene expression through DNA methylation
Gene and protein expression, including proteins involved in photosynthesis, stress response, secondary metabolism	*Arabidopsis* [163,164], bitter melon [165], blue sage [166], industrial hemp [167], maize [168], soybean [94,96], sunflower [58,169,170], tomato [171,172,173], wheat [81,174,175]	Changed expression and amounts of proteins in growing plants
Enzyme activities	*Arabidopsis* [87], artichoke [60], *A. fridae* [176], green chiretta [69], lemon balm [177], maize [178,179,180], pea [18], pepper [181], rice [182], soybean [94,96], sweet basil [183], tomato [172,184], wheat [89,98,185,186]	Changes in plant metabolism and antioxidant defense
Amount of phytohormones in plants	Maize [108], pea [71], tomato [173]	
Content of photosynthetic pigments	carrot [91], wheat [63,98,185,187,188], Norway spruce [70], maize [108,180], rice [93,178], soybean [189], spinach [147], tomato [172]	Improved growth due to up-regulated photosynthesis.
Activity or efficiency of photosynthesis	common buckwheat [109], maize [179], rice [182], pea [121], purple coneflower [190], soybean [189], sunflower [81], wheat [191]	
Secondary metabolism	coriander [92], brown rice [56,79], buckwheat [109], industrial hemp [48], maize [180,192], Norway spruce [70], purple coneflower [190], rapeseed [118], red clover [54,148,193], soybean [94], spinach [25], wheat [188,192]	Increased content of secondary metabolites is important for establishment of seedlings, plant fitness, stress resistance, communication with microorganisms.
Communication with microorganisms	*Arabidopsis* [194], sunflower [170], soybean [94], red clover [148]	Changed interactions with pathogens and beneficial microorganisms
Plant growth for the entire vegetation period and production yield	*Arabidopsis* [85], black mulberry [47], common buckwheat [109], garlic [195], industrial hemp [48], maize [179,196,197], Norway spruce [113], purple coneflower [190], peanut [198], red clover [54], rhododendron [47], tomato [40,171,199], wheat [63,191,192,197]	Improved plant growth for longer period of time. Persistent effects show the potential of NTP treatment for Plasma in Agriculture

## Data Availability

Not applicable.
